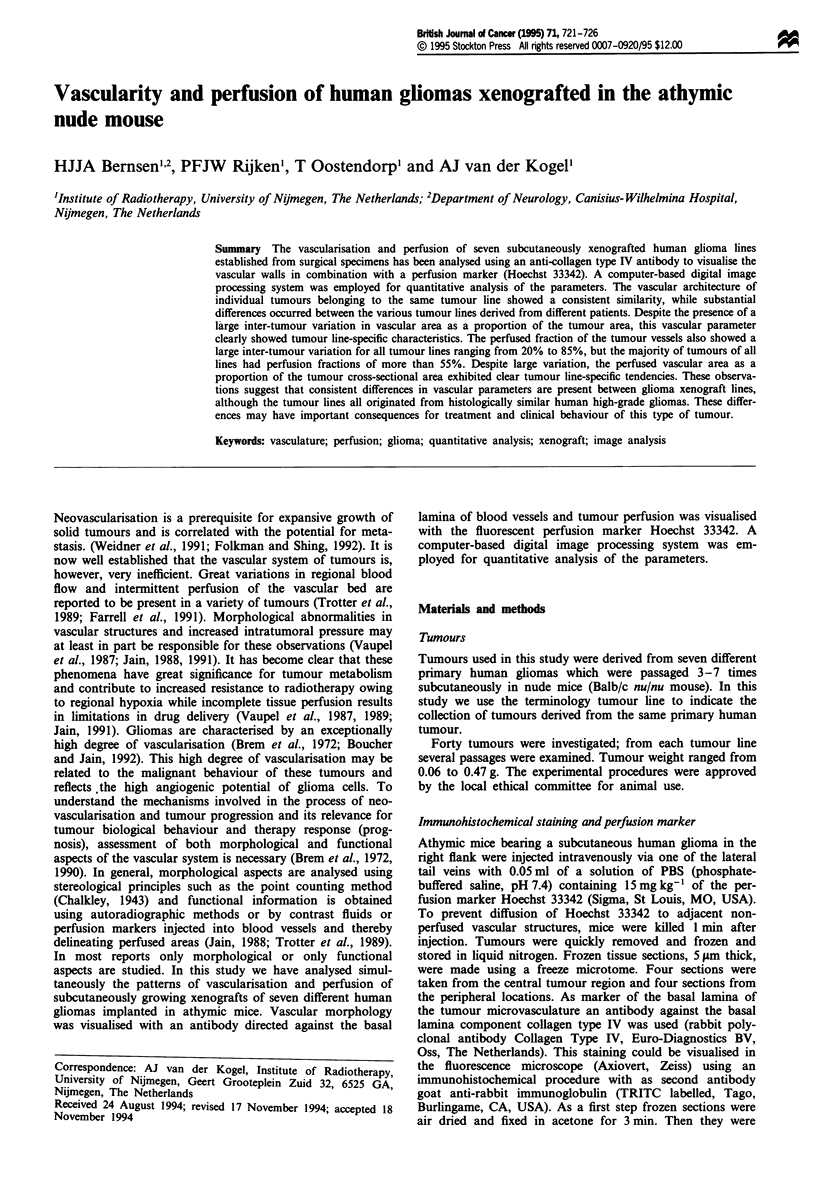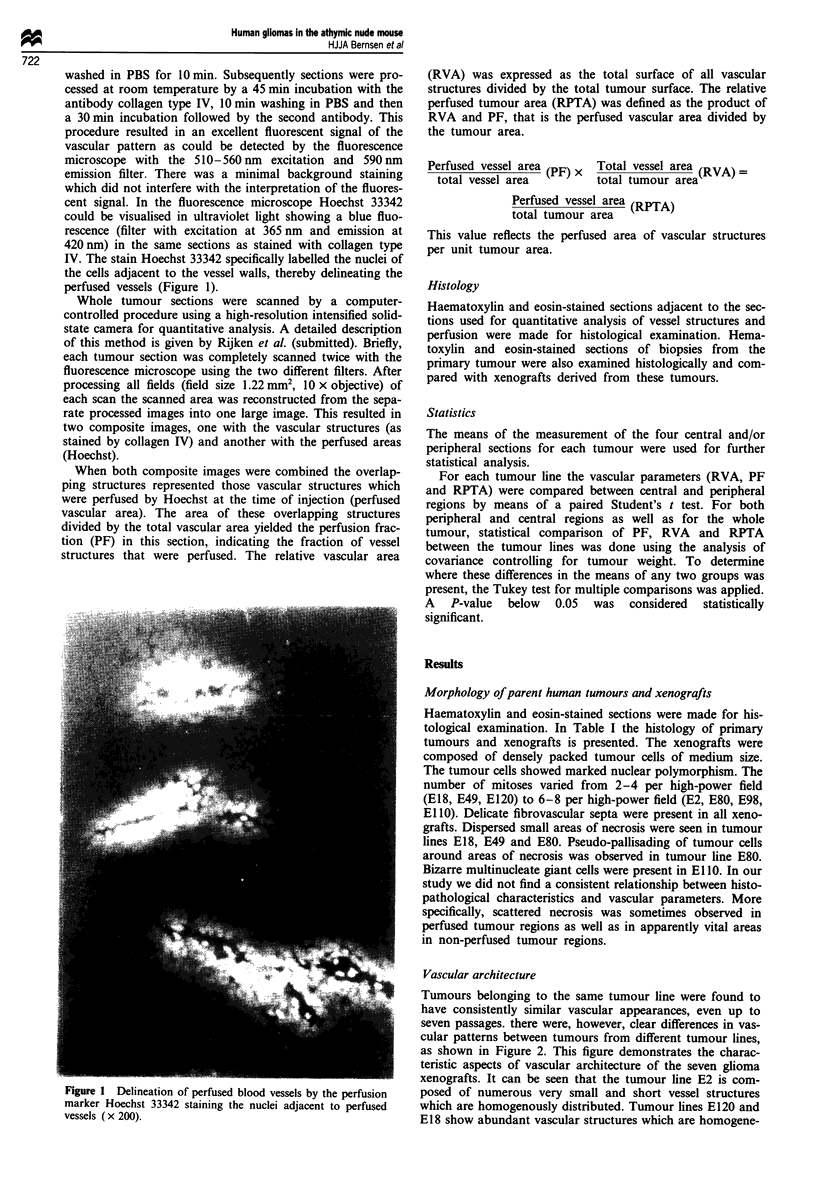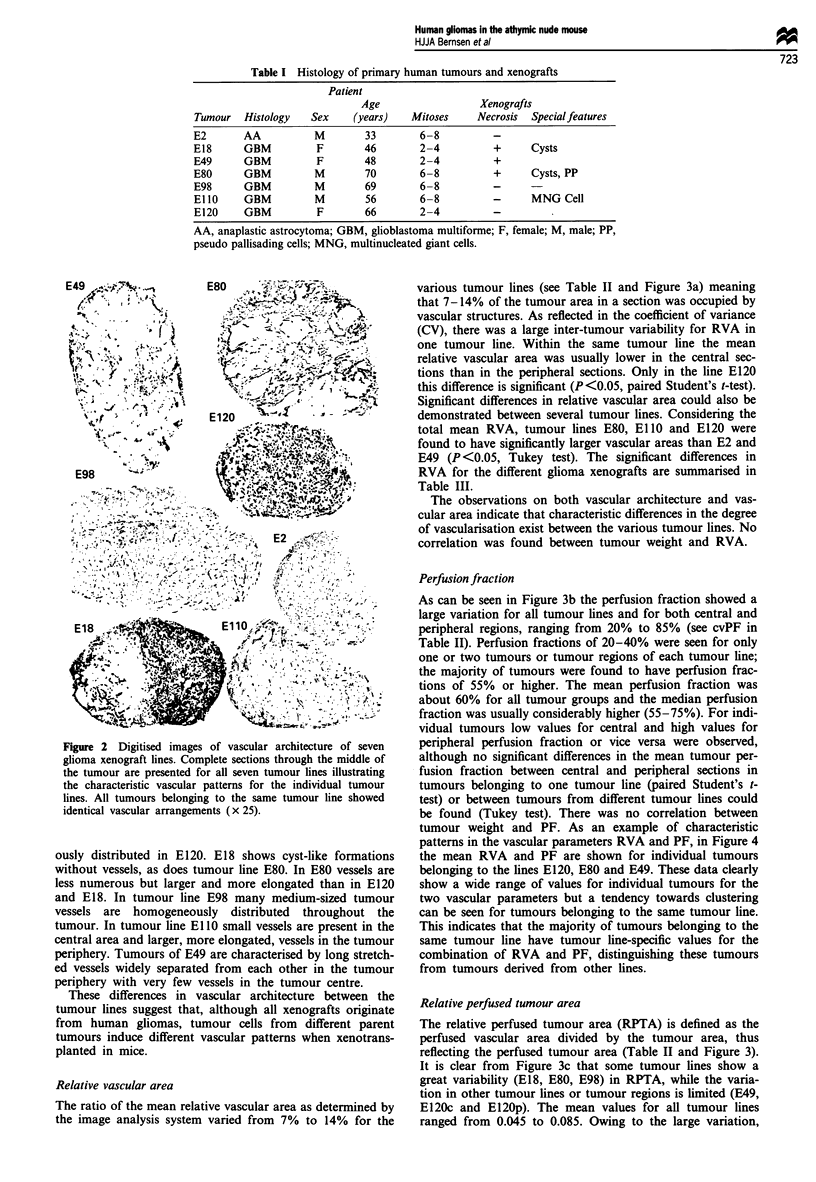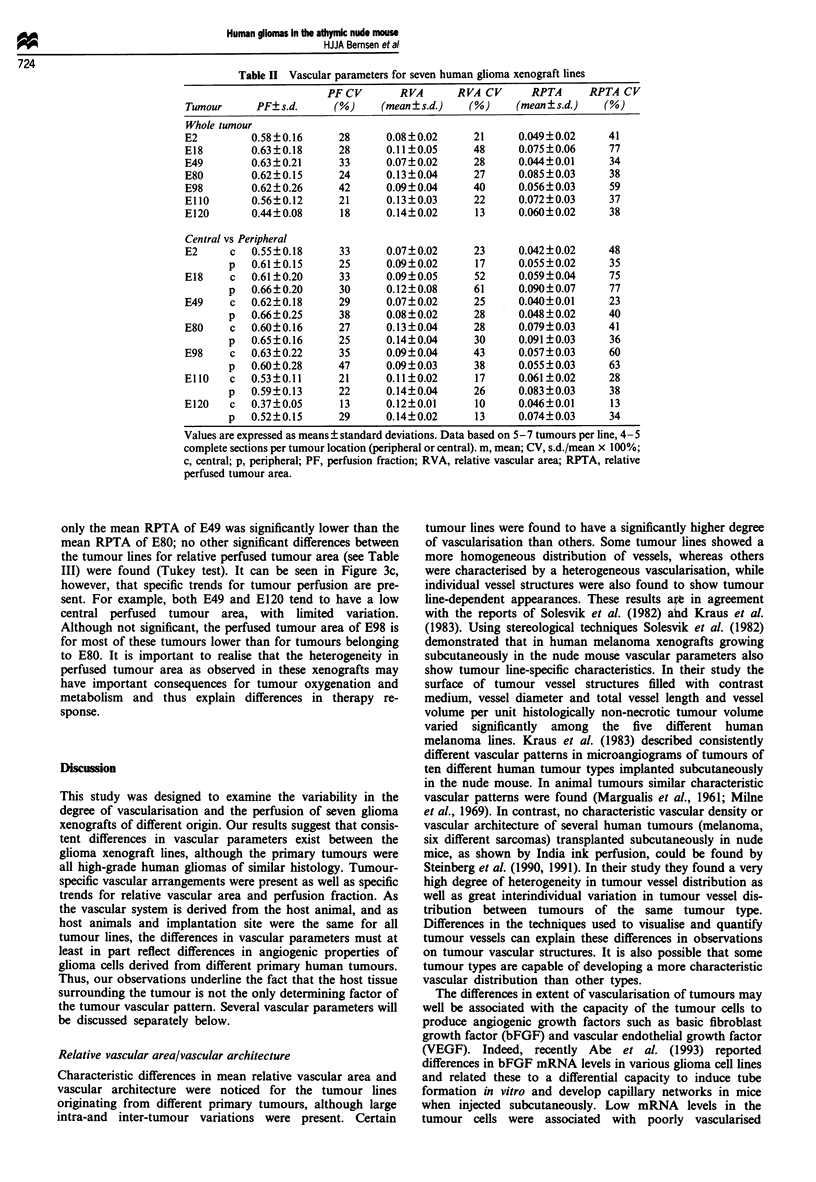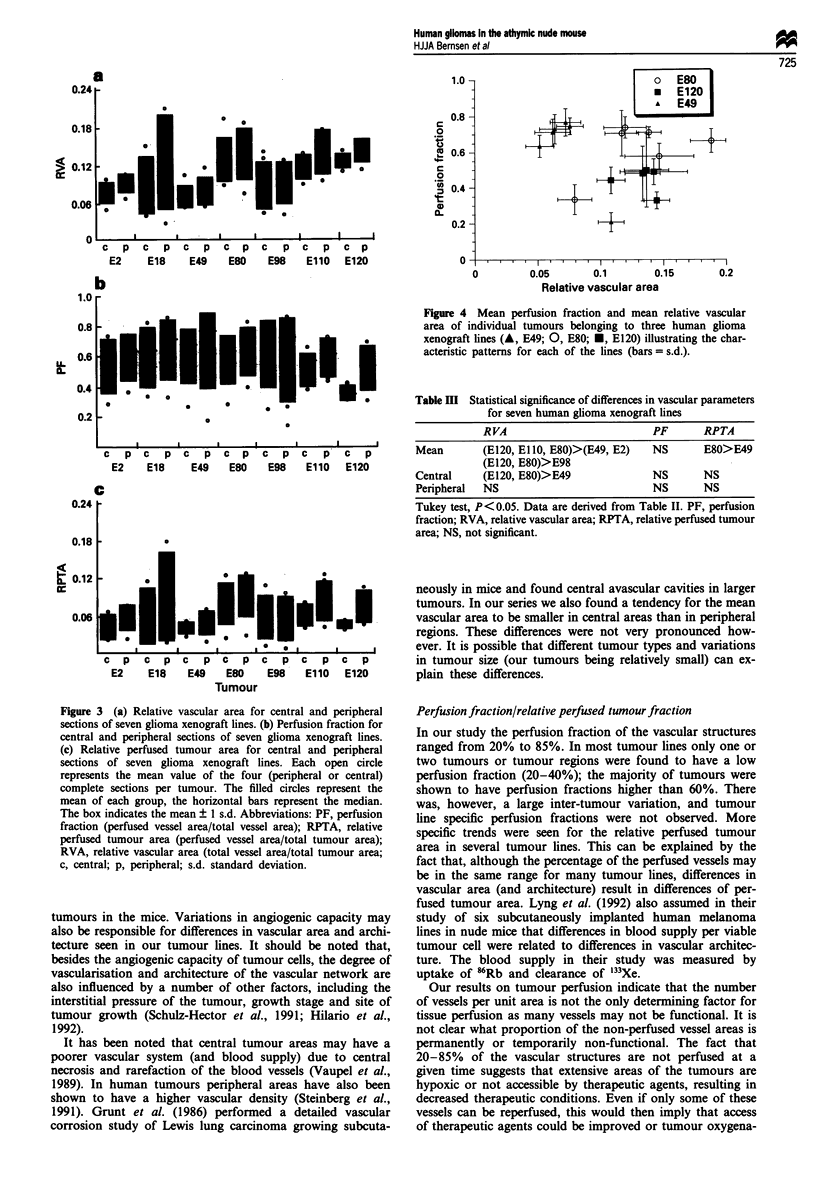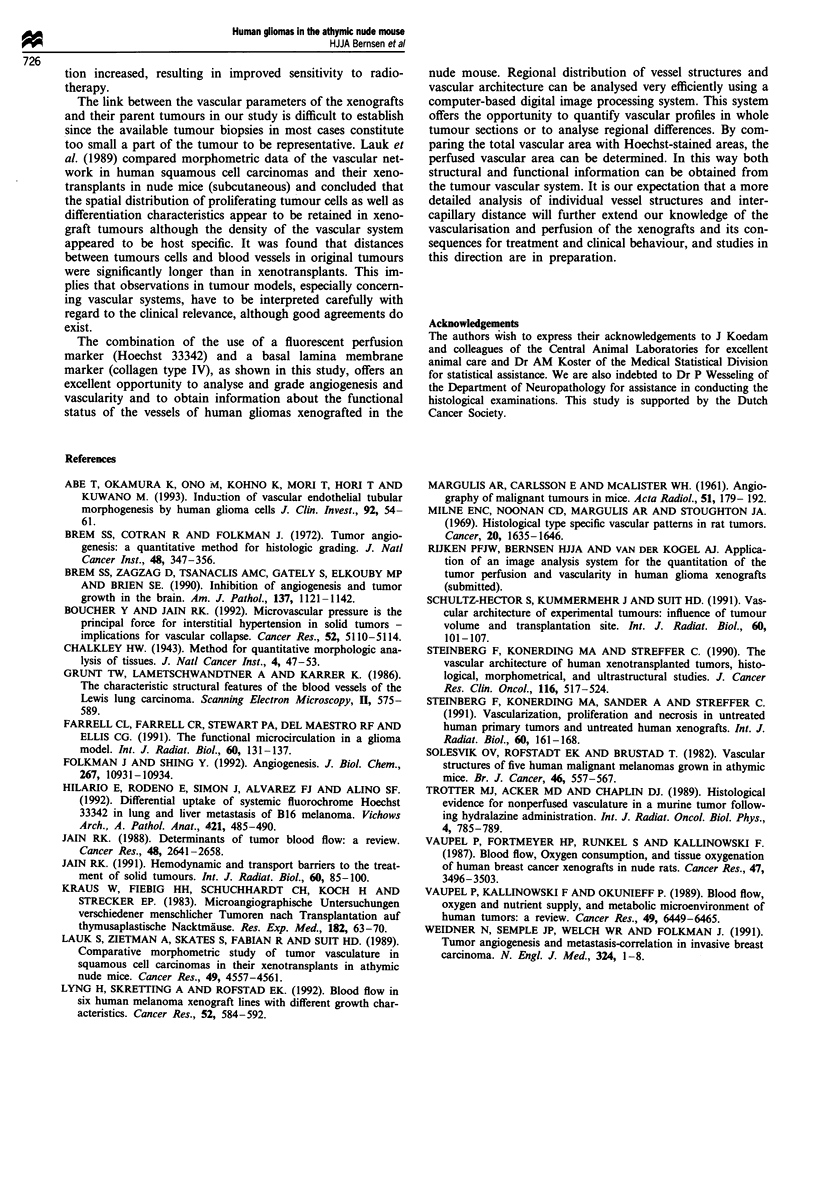# Vascularity and perfusion of human gliomas xenografted in the athymic nude mouse.

**DOI:** 10.1038/bjc.1995.141

**Published:** 1995-04

**Authors:** H. J. Bernsen, P. F. Rijken, T. Oostendorp, A. J. van der Kogel

**Affiliations:** Institute of Radiotherapy, University of Nijmegen, The Netherlands.

## Abstract

**Images:**


British Journal of Cancer (1995) 71, 721-726
© 1995 Stockton Press All rights reserved 0007-0920/95 $12.00

### Vascularity and perfusion of human gliomas xenografted in the athymic nude mouse

HJJA Bernsen[1,2], PFJW Rijken[1], T Oostendorp[1] and AJ van der Kogel[1]

[1]*Institute of Radiotherapy, University of Nijmegen, The Netherlands;* [2]*Department of Neurology, Canisius-Wilhelmina Hospital, Nijmegen, The Netherlands*

**Summary** The vascularisation and perfusion of seven subcutaneously xenografted human glioma lines established from surgical specimens has been analysed using an anti-collagen type IV antibody to visualise the vascular walls in combination with a perfusion marker (Hoechst 33342). A computer-based digital image processing system was employed for quantitative analysis of the parameters. The vascular architecture of individual tumours belonging to the same tumour line showed a consistent similarity, while substantial differences occurred between the various tumour lines derived from different patients. Despite the presence of a large inter-tumour variation in vascular area as a proportion of the tumour area, this vascular parameter clearly showed tumour line-specific characteristics. The perfused fraction of the tumour vessels also showed a large inter-tumour variation for all tumour lines ranging from 20% to 85%, but the majority of tumours of all lines had perfusion fractions of more than 55%. Despite large variation, the perfused vascular area as a proportion of the tumour cross-sectional area exhibited clear tumour line-specific tendencies. These observations suggest that consistent differences in vascular parameters are present between glioma xenograft lines, although the tumour lines all originated from histologically similar human high-grade gliomas. These differences may have important consequences for treatment and clinical behaviour of this type of tumour.

**Keywords:** vasculature; perfusion; glioma; quantitative analysis; xenograft; image analysis

Neovascularisation is a prerequisite for expansive growth of solid tumours and is correlated with the potential for metastasis. (Weidner *et al.*, 1991; Folkman and Shing, 1992). It is now well established that the vascular system of tumours is, however, very inefficient. Great variations in regional blood flow and intermittent perfusion of the vascular bed are reported to be present in a variety of tumours (Trotter *et al.*, 1989; Farrell *et al.*, 1991). Morphological abnormalities in vascular structures and increased intratumoral pressure may at least in part be responsible for these observations (Vaupel *et al.*, 1987; Jain, 1988, 1991). It has become clear that these phenomena have great significance for tumour metabolism and contribute to increased resistance to radiotherapy owing to regional hypoxia while incomplete tissue perfusion results in limitations in drug delivery (Vaupel *et al.*, 1987, 1989; Jain, 1991). Gliomas are characterised by an exceptionally high degree of vascularisation (Brem *et al.*, 1972; Boucher and Jain, 1992). This high degree of vascularisation may be related to the malignant behaviour of these tumours and reflects the high angiogenic potential of glioma cells. To understand the mechanisms involved in the process of neovascularisation and tumour progression and its relevance for tumour biological behaviour and therapy response (prognosis), assessment of both morphological and functional aspects of the vascular system is necessary (Brem *et al.*, 1972, 1990). In general, morphological aspects are analysed using stereological principles such as the point counting method (Chalkley, 1943) and functional information is obtained using autoradiographic methods or by contrast fluids or perfusion markers injected into blood vessels and thereby delineating perfused areas (Jain, 1988; Trotter *et al.*, 1989). In most reports only morphological or only functional aspects are studied. In this study we have analysed simultaneously the patterns of vascularisation and perfusion of subcutaneously growing xenografts of seven different human gliomas implanted in athymic mice. Vascular morphology was visualised with an antibody directed against the basal lamina of blood vessels and tumour perfusion was visualised with the fluorescent perfusion marker Hoechst 33342. A computer-based digital image processing system was employed for quantitative analysis of the parameters.

Correspondence: AJ van der Kogel, Institute of Radiotherapy, University of Nijmegen, Geert Grooteplein Zuid 32, 6525 GA, Nijmegen, The Netherlands
Received 24 August 1994; revised 17 November 1994; accepted 18 November 1994

### Materials and methods

### *Tumours*

Tumours used in this study were derived from seven different primary human gliomas which were passaged 3-7 times subcutaneously in nude mice (Balb/c *nu/nu* mouse). In this study we use the terminology tumour line to indicate the collection of tumours derived from the same primary human tumour.

Forty tumours were investigated; from each tumour line several passages were examined. Tumour weight ranged from 0.06 to 0.47 g. The experimental procedures were approved by the local ethical committee for animal use.

### *Immunohistochemical staining and perfusion marker*

Athymic mice bearing a subcutaneous human glioma in the right flank were injected intravenously via one of the lateral tail veins with 0.05 ml of a solution of PBS (phosphate-buffered saline, pH 7.4) containing 15 mg $kg^{-1}$ of the perfusion marker Hoechst 33342 (Sigma, St Louis, MO, USA). To prevent diffusion of Hoechst 33342 to adjacent non-perfused vascular structures, mice were killed 1 min after injection. Tumours were quickly removed and frozen and stored in liquid nitrogen. Frozen tissue sections, 5 μm thick, were made using a freeze microtome. Four sections were taken from the central tumour region and four sections from the peripheral locations. As marker of the basal lamina of the tumour microvasculature an antibody against the basal lamina component collagen type IV was used (rabbit polyclonal antibody Collagen Type IV, Euro-Diagnostics BV, Oss, The Netherlands). This staining could be visualised in the fluorescence microscope (Axiovert, Zeiss) using an immunohistochemical procedure with as second antibody goat anti-rabbit immunoglobulin (TRITC labelled, Tago, Burlingame, CA, USA). As a first step frozen sections were air dried and fixed in acetone for 3 min. Then they were washed in PBS for 10 min. Subsequently sections were processed at room temperature by a 45 min incubation with the antibody collagen type IV, 10 min washing in PBS and then a 30 min incubation followed by the second antibody. This procedure resulted in an excellent fluorescent signal of the vascular pattern as could be detected by the fluorescence microscope with the 510–560 nm excitation and 590 nm emission filter. There was a minimal background staining which did not interfere with the interpretation of the fluorescent signal. In the fluorescence microscope Hoechst 33342 could be visualised in ultraviolet light showing a blue fluorescence (filter with excitation at 365 nm and emission at 420 nm) in the same sections as stained with collagen type IV. The stain Hoechst 33342 specifically labelled the nuclei of the cells adjacent to the vessel walls, thereby delineating the perfused vessels (Figure 1).

Whole tumour sections were scanned by a computer-controlled procedure using a high-resolution intensified solid-state camera for quantitative analysis. A detailed description of this method is given by Rijken *et al.* (submitted). Briefly, each tumour section was completely scanned twice with the fluorescence microscope using the two different filters. After processing all fields (field size 1.22 $mm^2$, 10 × objective) of each scan the scanned area was reconstructed from the separate processed images into one large image. This resulted in two composite images, one with the vascular structures (as stained by collagen IV) and another with the perfused areas (Hoechst).

When both composite images were combined the overlapping structures represented those vascular structures which were perfused by Hoechst at the time of injection (perfused vascular area). The area of these overlapping structures divided by the total vascular area yielded the perfusion fraction (PF) in this section, indicating the fraction of vessel structures that were perfused. The relative vascular area (RVA) was expressed as the total surface of all vascular structures divided by the total tumour surface. The relative perfused tumour area (RPTA) was defined as the product of RVA and PF, that is the perfused vascular area divided by the tumour area.

$$\frac{\text{Perfused vessel area}}{\text{total vessel area}}(\text{PF}) \times \frac{\text{Total vessel area}}{\text{total tumour area}}(\text{RVA}) = \frac{\text{Perfused vessel area}}{\text{total tumour area}}(\text{RPTA})$$

This value reflects the perfused area of vascular structures per unit tumour area.

### *Histology*

Haematoxylin and eosin-stained sections adjacent to the sections used for quantitative analysis of vessel structures and perfusion were made for histological examination. Hematoxylin and eosin-stained sections of biopsies from the primary tumour were also examined histologically and compared with xenografts derived from these tumours.

### *Statistics*

The means of the measurement of the four central and/or peripheral sections for each tumour were used for further statistical analysis.

For each tumour line the vascular parameters (RVA, PF and RPTA) were compared between central and peripheral regions by means of a paired Student's $t$ test. For both peripheral and central regions as well as for the whole tumour, statistical comparison of PF, RVA and RPTA between the tumour lines was done using the analysis of covariance controlling for tumour weight. To determine where these differences in the means of any two groups was present, the Tukey test for multiple comparisons was applied. A $P$-value below 0.05 was considered statistically significant.

**Figure 1** Delineation of perfused blood vessels by the perfusion marker Hoechst 33342 staining the nuclei adjacent to perfused vessels (× 200).

### Results

### *Morphology of parent human tumours and xenografts*

Haematoxylin and eosin-stained sections were made for histological examination. In Table I the histology of primary tumours and xenografts is presented. The xenografts were composed of densely packed tumour cells of medium size. The tumour cells showed marked nuclear polymorphism. The number of mitoses varied from 2–4 per high-power field (E18, E49, E120) to 6–8 per high-power field (E2, E80, E98, E110). Delicate fibrovascular septa were present in all xenografts. Dispersed small areas of necrosis were seen in tumour lines E18, E49 and E80. Pseudo-pallisading of tumour cells around areas of necrosis was observed in tumour line E80. Bizarre multinucleate giant cells were present in E110. In our study we did not find a consistent relationship between histopathological characteristics and vascular parameters. More specifically, scattered necrosis was sometimes observed in perfused tumour regions as well as in apparently vital areas in non-perfused tumour regions.

### *Vascular architecture*

Tumours belonging to the same tumour line were found to have consistently similar vascular appearances, even up to seven passages. there were, however, clear differences in vascular patterns between tumours from different tumour lines, as shown in Figure 2. This figure demonstrates the characteristic aspects of vascular architecture of the seven glioma xenografts. It can be seen that the tumour line E2 is composed of numerous very small and short vessel structures which are homogenously distributed. Tumour lines E120 and E18 show abundant vascular structures which are homogene-

**Table I** Histology of primary human tumours and xenografts

| | Patient | | | | Xenografts | |
|---|---|---|---|---|---|---|
| *Tumour* | *Histology* | *Sex* | *Age (years)* | *Mitoses* | *Necrosis* | *Special features* |
| E2 | AA | M | 33 | 6–8 | – | |
| E18 | GBM | F | 46 | 2–4 | + | Cysts |
| E49 | GBM | F | 48 | 2–4 | + | |
| E80 | GBM | M | 70 | 6–8 | + | Cysts, PP |
| E98 | GBM | M | 69 | 6–8 | – | — |
| E110 | GBM | M | 56 | 6–8 | – | MNG Cell |
| E120 | GBM | F | 66 | 2–4 | – | |

AA, anaplastic astrocytoma; GBM, glioblastoma multiforme; F, female; M, male; PP, pseudo pallisading cells; MNG, multinucleated giant cells.

**Figure 2** Digitised images of vascular architecture of seven glioma xenograft lines. Complete sections through the middle of the tumour are presented for all seven tumour lines illustrating the characteristic vascular patterns for the individual tumour lines. All tumours belonging to the same tumour line showed identical vascular arrangements (×25).

ously distributed in E120. E18 shows cyst-like formations without vessels, as does tumour line E80. In E80 vessels are less numerous but larger and more elongated than in E120 and E18. In tumour line E98 many medium-sized tumour vessels are homogeneously distributed throughout the tumour. In tumour line E110 small vessels are present in the central area and larger, more elongated, vessels in the tumour periphery. Tumours of E49 are characterised by long stretched vessels widely separated from each other in the tumour periphery with very few vessels in the tumour centre.

These differences in vascular architecture between the tumour lines suggest that, although all xenografts originate from human gliomas, tumour cells from different parent tumours induce different vascular patterns when xenotransplanted in mice.

### *Relative vascular area*

The ratio of the mean relative vascular area as determined by the image analysis system varied from 7% to 14% for the various tumour lines (see Table II and Figure 3a) meaning that 7–14% of the tumour area in a section was occupied by vascular structures. As reflected in the coefficient of variance (CV), there was a large inter-tumour variability for RVA in one tumour line. Within the same tumour line the mean relative vascular area was usually lower in the central sections than in the peripheral sections. Only in the line E120 this difference is significant ($P<0.05$, paired Student's $t$-test). Significant differences in relative vascular area could also be demonstrated between several tumour lines. Considering the total mean RVA, tumour lines E80, E110 and E120 were found to have significantly larger vascular areas than E2 and E49 ($P<0.05$, Tukey test). The significant differences in RVA for the different glioma xenografts are summarised in Table III.

The observations on both vascular architecture and vascular area indicate that characteristic differences in the degree of vascularisation exist between the various tumour lines. No correlation was found between tumour weight and RVA.

### *Perfusion fraction*

As can be seen in Figure 3b the perfusion fraction showed a large variation for all tumour lines and for both central and peripheral regions, ranging from 20% to 85% (see cvPF in Table II). Perfusion fractions of 20–40% were seen for only one or two tumours or tumour regions of each tumour line; the majority of tumours were found to have perfusion fractions of 55% or higher. The mean perfusion fraction was about 60% for all tumour groups and the median perfusion fraction was usually considerably higher (55–75%). For individual tumours low values for central and high values for peripheral perfusion fraction or vice versa were observed, although no significant differences in the mean tumour perfusion fraction between central and peripheral sections in tumours belonging to one tumour line (paired Student's $t$-test) or between tumours from different tumour lines could be found (Tukey test). There was no correlation between tumour weight and PF. As an example of characteristic patterns in the vascular parameters RVA and PF, in Figure 4 the mean RVA and PF are shown for individual tumours belonging to the lines E120, E80 and E49. These data clearly show a wide range of values for individual tumours for the two vascular parameters but a tendency towards clustering can be seen for tumours belonging to the same tumour line. This indicates that the majority of tumours belonging to the same tumour line have tumour line-specific values for the combination of RVA and PF, distinguishing these tumours from tumours derived from other lines.

### *Relative perfused tumour area*

The relative perfused tumour area (RPTA) is defined as the perfused vascular area divided by the tumour area, thus reflecting the perfused tumour area (Table II and Figure 3). It is clear from Figure 3c that some tumour lines show a great variability (E18, E80, E98) in RPTA, while the variation in other tumour lines or tumour regions is limited (E49, E120c and E120p). The mean values for all tumour lines ranged from 0.045 to 0.085. Owing to the large variation,

Table II Vascular parameters for seven human glioma xenograft lines

| Tumour | | PF ± s.d. | PF CV (%) | RVA (mean ± s.d.) | RVA CV (%) | RPTA (mean ± s.d.) | RPTA CV (%) |
|---|---|---|---|---|---|---|---|
| *Whole tumour* | | | | | | | |
| E2 | | 0.58 ± 0.16 | 28 | 0.08 ± 0.02 | 21 | 0.049 ± 0.02 | 41 |
| E18 | | 0.63 ± 0.18 | 28 | 0.11 ± 0.05 | 48 | 0.075 ± 0.06 | 77 |
| E49 | | 0.63 ± 0.21 | 33 | 0.07 ± 0.02 | 28 | 0.044 ± 0.01 | 34 |
| E80 | | 0.62 ± 0.15 | 24 | 0.13 ± 0.04 | 27 | 0.085 ± 0.03 | 38 |
| E98 | | 0.62 ± 0.26 | 42 | 0.09 ± 0.04 | 40 | 0.056 ± 0.03 | 59 |
| E110 | | 0.56 ± 0.12 | 21 | 0.13 ± 0.03 | 22 | 0.072 ± 0.03 | 37 |
| E120 | | 0.44 ± 0.08 | 18 | 0.14 ± 0.02 | 13 | 0.060 ± 0.02 | 38 |
| *Central* vs *Peripheral* | | | | | | | |
| E2 | c | 0.55 ± 0.18 | 33 | 0.07 ± 0.02 | 23 | 0.042 ± 0.02 | 48 |
| | p | 0.61 ± 0.15 | 25 | 0.09 ± 0.02 | 17 | 0.055 ± 0.02 | 35 |
| E18 | c | 0.61 ± 0.20 | 33 | 0.09 ± 0.05 | 52 | 0.059 ± 0.04 | 75 |
| | p | 0.66 ± 0.20 | 30 | 0.12 ± 0.08 | 61 | 0.090 ± 0.07 | 77 |
| E49 | c | 0.62 ± 0.18 | 29 | 0.07 ± 0.02 | 25 | 0.040 ± 0.01 | 23 |
| | p | 0.66 ± 0.25 | 38 | 0.08 ± 0.02 | 28 | 0.048 ± 0.02 | 40 |
| E80 | c | 0.60 ± 0.16 | 27 | 0.13 ± 0.04 | 28 | 0.079 ± 0.03 | 41 |
| | p | 0.65 ± 0.16 | 25 | 0.14 ± 0.04 | 30 | 0.091 ± 0.03 | 36 |
| E98 | c | 0.63 ± 0.22 | 35 | 0.09 ± 0.04 | 43 | 0.057 ± 0.03 | 60 |
| | p | 0.60 ± 0.28 | 47 | 0.09 ± 0.03 | 38 | 0.055 ± 0.03 | 63 |
| E110 | c | 0.53 ± 0.11 | 21 | 0.11 ± 0.02 | 17 | 0.061 ± 0.02 | 28 |
| | p | 0.59 ± 0.13 | 22 | 0.14 ± 0.04 | 26 | 0.083 ± 0.03 | 38 |
| E120 | c | 0.37 ± 0.05 | 13 | 0.12 ± 0.01 | 10 | 0.046 ± 0.01 | 13 |
| | p | 0.52 ± 0.15 | 29 | 0.14 ± 0.02 | 13 | 0.074 ± 0.03 | 34 |

Values are expressed as means ± standard deviations. Data based on 5–7 tumours per line, 4–5 complete sections per tumour location (peripheral or central). m, mean; CV, s.d./mean × 100%; c, central; p, peripheral; PF, perfusion fraction; RVA, relative vascular area; RPTA, relative perfused tumour area.

only the mean RPTA of E49 was significantly lower than the mean RPTA of E80; no other significant differences between the tumour lines for relative perfused tumour area (see Table III) were found (Tukey test). It can be seen in Figure 3c, however, that specific trends for tumour perfusion are present. For example, both E49 and E120 tend to have a low central perfused tumour area, with limited variation. Although not significant, the perfused tumour area of E98 is for most of these tumours lower than for tumours belonging to E80. It is important to realise that the heterogeneity in perfused tumour area as observed in these xenografts may have important consequences for tumour oxygenation and metabolism and thus explain differences in therapy response.

### Discussion

This study was designed to examine the variability in the degree of vascularisation and the perfusion of seven glioma xenografts of different origin. Our results suggest that consistent differences in vascular parameters exist between the glioma xenograft lines, although the primary tumours were all high-grade human gliomas of similar histology. Tumour-specific vascular arrangements were present as well as specific trends for relative vascular area and perfusion fraction. As the vascular system is derived from the host animal, and as host animals and implantation site were the same for all tumour lines, the differences in vascular parameters must at least in part reflect differences in angiogenic properties of glioma cells derived from different primary human tumours. Thus, our observations underline the fact that the host tissue surrounding the tumour is not the only determining factor of the tumour vascular pattern. Several vascular parameters will be discussed separately below.

### *Relative vascular area/vascular architecture*

Characteristic differences in mean relative vascular area and vascular architecture were noticed for the tumour lines originating from different primary tumours, although large intra-and inter-tumour variations were present. Certain tumour lines were found to have a significantly higher degree of vascularisation than others. Some tumour lines showed a more homogeneous distribution of vessels, whereas others were characterised by a heterogeneous vascularisation, while individual vessel structures were also found to show tumour line-dependent appearances. These results are in agreement with the reports of Solesvik *et al.* (1982) and Kraus *et al.* (1983). Using stereological techniques Solesvik *et al.* (1982) demonstrated that in human melanoma xenografts growing subcutaneously in the nude mouse vascular parameters also show tumour line-specific characteristics. In their study the surface of tumour vessel structures filled with contrast medium, vessel diameter and total vessel length and vessel volume per unit histologically non-necrotic tumour volume varied significantly among the five different human melanoma lines. Kraus *et al.* (1983) described consistently different vascular patterns in microangiograms of tumours of ten different human tumour types implanted subcutaneously in the nude mouse. In animal tumours similar characteristic vascular patterns were found (Margualis *et al.*, 1961; Milne *et al.*, 1969). In contrast, no characteristic vascular density or vascular architecture of several human tumours (melanoma, six different sarcomas) transplanted subcutaneously in nude mice, as shown by India ink perfusion, could be found by Steinberg *et al.* (1990, 1991). In their study they found a very high degree of heterogeneity in tumour vessel distribution as well as great interindividual variation in tumour vessel distribution between tumours of the same tumour type. Differences in the techniques used to visualise and quantify tumour vessels can explain these differences in observations on tumour vascular structures. It is also possible that some tumour types are capable of developing a more characteristic vascular distribution than other types.

The differences in extent of vascularisation of tumours may well be associated with the capacity of the tumour cells to produce angiogenic growth factors such as basic fibroblast growth factor (bFGF) and vascular endothelial growth factor (VEGF). Indeed, recently Abe *et al.* (1993) reported differences in bFGF mRNA levels in various glioma cell lines and related these to a differential capacity to induce tube formation *in vitro* and develop capillary networks in mice when injected subcutaneously. Low mRNA levels in the tumour cells were associated with poorly vascularised

**Figure 3** (**a**) Relative vascular area for central and peripheral sections of seven glioma xenograft lines. (**b**) Perfusion fraction for central and peripheral sections of seven glioma xenograft lines. (**c**) Relative perfused tumour area for central and peripheral sections of seven glioma xenograft lines. Each open circle represents the mean value of the four (peripheral or central) complete sections per tumour. The filled circles represent the mean of each group, the horizontal bars represent the median. The box indicates the mean ± 1 s.d. Abbreviations: PF, perfusion fraction (perfused vessel area/total vessel area); RPTA, relative perfused tumour area (perfused vessel area/total tumour area); RVA, relative vascular area (total vessel area/total tumour area; c, central; p, peripheral; s.d. standard deviation.

tumours in the mice. Variations in angiogenic capacity may also be responsible for differences in vascular area and architecture seen in our tumour lines. It should be noted that, besides the angiogenic capacity of tumour cells, the degree of vascularisation and architecture of the vascular network are also influenced by a number of other factors, including the interstitial pressure of the tumour, growth stage and site of tumour growth (Schulz-Hector *et al.*, 1991; Hilario *et al.*, 1992).

It has been noted that central tumour areas may have a poorer vascular system (and blood supply) due to central necrosis and rarefaction of the blood vessels (Vaupel *et al.*, 1989). In human tumours peripheral areas have also been shown to have a higher vascular density (Steinberg *et al.*, 1991). Grunt *et al.* (1986) performed a detailed vascular corrosion study of Lewis lung carcinoma growing subcutaneously in mice and found central avascular cavities in larger tumours. In our series we also found a tendency for the mean vascular area to be smaller in central areas than in peripheral regions. These differences were not very pronounced however. It is possible that different tumour types and variations in tumour size (our tumours being relatively small) can explain these differences.

**Figure 4** Mean perfusion fraction and mean relative vascular area of individual tumours belonging to three human glioma xenograft lines (▲, E49; O, E80; ■, E120) illustrating the characteristic patterns for each of the lines (bars = s.d.).

**Table III** Statistical significance of differences in vascular parameters for seven human glioma xenograft lines

| | *RVA* | *PF* | *RPTA* |
|---|---|---|---|
| Mean | (E120, E110, E80)>(E49, E2)<br>(E120, E80)>E98 | NS | E80>E49 |
| Central | (E120, E80)>E49 | NS | NS |
| Peripheral | NS | NS | NS |

Tukey test, $P<0.05$. Data are derived from Table II. PF, perfusion fraction; RVA, relative vascular area; RPTA, relative perfused tumour area; NS, not significant.

*Perfusion fraction/relative perfused tumour fraction*

In our study the perfusion fraction of the vascular structures ranged from 20% to 85%. In most tumour lines only one or two tumours or tumour regions were found to have a low perfusion fraction (20–40%); the majority of tumours were shown to have perfusion fractions higher than 60%. There was, however, a large inter-tumour variation, and tumour line specific perfusion fractions were not observed. More specific trends were seen for the relative perfused tumour area in several tumour lines. This can be explained by the fact that, although the percentage of the perfused vessels may be in the same range for many tumour lines, differences in vascular area (and architecture) result in differences of perfused tumour area. Lyng *et al.* (1992) also assumed in their study of six subcutaneously implanted human melanoma lines in nude mice that differences in blood supply per viable tumour cell were related to differences in vascular architecture. The blood supply in their study was measured by uptake of $^{86}$Rb and clearance of $^{133}$Xe.

Our results on tumour perfusion indicate that the number of vessels per unit area is not the only determining factor for tissue perfusion as many vessels may not be functional. It is not clear what proportion of the non-perfused vessel areas is permanently or temporarily non-functional. The fact that 20–85% of the vascular structures are not perfused at a given time suggests that extensive areas of the tumours are hypoxic or not accessible by therapeutic agents, resulting in decreased therapeutic conditions. Even if only some of these vessels can be reperfused, this would then imply that access of therapeutic agents could be improved or tumour oxygenation increased, resulting in improved sensitivity to radiotherapy.

The link between the vascular parameters of the xenografts and their parent tumours in our study is difficult to establish since the available tumour biopsies in most cases constitute too small a part of the tumour to be representative. Lauk et al. (1989) compared morphometric data of the vascular network in human squamous cell carcinomas and their xenotransplants in nude mice (subcutaneous) and concluded that the spatial distribution of proliferating tumour cells as well as differentiation characteristics appear to be retained in xenograft tumours although the density of the vascular system appeared to be host specific. It was found that distances between tumours cells and blood vessels in original tumours were significantly longer than in xenotransplants. This implies that observations in tumour models, especially concerning vascular systems, have to be interpreted carefully with regard to the clinical relevance, although good agreements do exist.

The combination of the use of a fluorescent perfusion marker (Hoechst 33342) and a basal lamina membrane marker (collagen type IV), as shown in this study, offers an excellent opportunity to analyse and grade angiogenesis and vascularity and to obtain information about the functional status of the vessels of human gliomas xenografted in the nude mouse. Regional distribution of vessel structures and vascular architecture can be analysed very efficiently using a computer-based digital image processing system. This system offers the opportunity to quantify vascular profiles in whole tumour sections or to analyse regional differences. By comparing the total vascular area with Hoechst-stained areas, the perfused vascular area can be determined. In this way both structural and functional information can be obtained from the tumour vascular system. It is our expectation that a more detailed analysis of individual vessel structures and intercapillary distance will further extend our knowledge of the vascularisation and perfusion of the xenografts and its consequences for treatment and clinical behaviour, and studies in this direction are in preparation.

**Acknowledgements**
The authors wish to express their acknowledgements to J Koedam and colleagues of the Central Animal Laboratories for excellent animal care and Dr AM Koster of the Medical Statistical Division for statistical assistance. We are also indebted to Dr P Wesseling of the Department of Neuropathology for assistance in conducting the histological examinations. This study is supported by the Dutch Cancer Society.

**References**

ABE T, OKAMURA K, ONO M, KOHNO K, MORI T, HORI T AND KUWANO M. (1993). Induction of vascular endothelial tubular morphogenesis by human glioma cells *J. Clin. Invest.*, **92**, 54-61.

BREM SS, COTRAN R AND FOLKMAN J. (1972). Tumor angiogenesis: a quantitative method for histologic grading. *J. Natl Cancer Inst.*, **48**, 347-356.

BREM SS, ZAGZAG D, TSANACLIS AMC, GATELY S, ELKOUBY MP AND BRIEN SE. (1990). Inhibition of angiogenesis and tumor growth in the brain. *Am. J. Pathol.*, **137**, 1121-1142.

BOUCHER Y AND JAIN RK. (1992). Microvascular pressure is the principal force for interstitial hypertension in solid tumors - implications for vascular collapse. *Cancer Res.*, **52**, 5110-5114.

CHALKLEY HW. (1943). Method for quantitative morphologic analysis of tissues. *J. Natl Cancer Inst.*, **4**, 47-53.

GRUNT TW, LAMETSCHWANDTNER A AND KARRER K. (1986). The characteristic structural features of the blood vessels of the Lewis lung carcinoma. *Scanning Electron Microscopy*, **II**, 575-589.

FARRELL CL, FARRELL CR, STEWART PA, DEL MAESTRO RF AND ELLIS CG. (1991). The functional microcirculation in a glioma model. *Int. J. Radiat. Biol.*, **60**, 131-137.

FOLKMAN J AND SHING Y. (1992). Angiogenesis. *J. Biol. Chem.*, **267**, 10931-10934.

HILARIO E, RODENO E, SIMON J, ALVAREZ FJ AND ALINO SF. (1992). Differential uptake of systemic fluorochrome Hoechst 33342 in lung and liver metastasis of B16 melanoma. *Vichows Arch., A. Pathol. Anat.*, **421**, 485-490.

JAIN RK. (1988). Determinants of tumor blood flow: a review. *Cancer Res.*, **48**, 2641-2658.

JAIN RK. (1991). Hemodynamic and transport barriers to the treatment of solid tumours. *Int. J. Radiat. Biol.*, **60**, 85-100.

KRAUS W, FIEBIG HH, SCHUCHHARDT CH, KOCH H AND STRECKER EP. (1983). Microangiographische Untersuchungen verschiedener menschlicher Tumoren nach Transplantation auf thymusaplastische Nacktmäuse. *Res. Exp. Med.*, **182**, 63-70.

LAUK S, ZIETMAN A, SKATES S, FABIAN R AND SUIT HD. (1989). Comparative morphometric study of tumor vasculature in squamous cell carcinomas in their xenotransplants in athymic nude mice. *Cancer Res.*, **49**, 4557-4561.

LYNG H, SKRETTING A AND ROFSTAD EK. (1992). Blood flow in six human melanoma xenograft lines with different growth characteristics. *Cancer Res.*, **52**, 584-592.

MARGULIS AR, CARLSSON E AND MCALISTER WH. (1961). Angiography of malignant tumours in mice. *Acta Radiol.*, **51**, 179- 192.

MILNE ENC, NOONAN CD, MARGULIS AR AND STOUGHTON JA. (1969). Histological type specific vascular patterns in rat tumors. *Cancer*, **20**, 1635-1646.

RIJKEN PFJW, BERNSEN HJJA AND VAN DER KOGEL AJ. Application of an image analysis system for the quantitation of the tumor perfusion and vascularity in human glioma xenografts (submitted).

SCHULTZ-HECTOR S, KUMMERMEHR J AND SUIT HD. (1991). Vascular architecture of experimental tumours: influence of tumour volume and transplantation site. *Int. J. Radiat. Biol.*, **60**, 101-107.

STEINBERG F, KONERDING MA AND STREFFER C. (1990). The vascular architecture of human xenotransplanted tumors, histological, morphometrical, and ultrastructural studies. *J. Cancer Res. Clin. Oncol.*, **116**, 517-524.

STEINBERG F, KONERDING MA, SANDER A AND STREFFER C. (1991). Vascularization, proliferation and necrosis in untreated human primary tumors and untreated human xenografts. *Int. J. Radiat. Biol.*, **60**, 161-168.

SOLESVIK OV, ROFSTADT EK AND BRUSTAD T. (1982). Vascular structures of five human malignant melanomas grown in athymic mice. *Br. J. Cancer*, **46**, 557-567.

TROTTER MJ, ACKER MD AND CHAPLIN DJ. (1989). Histological evidence for nonperfused vasculature in a murine tumor following hydralazine administration. *Int. J. Radiat. Oncol. Biol. Phys.*, **4**, 785-789.

VAUPEL P, FORTMEYER HP, RUNKEL S AND KALLINOWSKI F. (1987). Blood flow, Oxygen consumption, and tissue oxygenation of human breast cancer xenografts in nude rats. *Cancer Res.*, **47**, 3496-3503.

VAUPEL P, KALLINOWSKI F AND OKUNIEFF P. (1989). Blood flow, oxygen and nutrient supply, and metabolic microenvironment of human tumors: a review. *Cancer Res.*, **49**, 6449-6465.

WEIDNER N, SEMPLE JP, WELCH WR AND FOLKMAN J. (1991). Tumor angiogenesis and metastasis-correlation in invasive breast carcinoma. *N. Engl. J. Med.*, **324**, 1-8.